# A humanized orthotopic tumor microenvironment alters the bone metastatic tropism of prostate cancer cells

**DOI:** 10.1038/s42003-021-02527-x

**Published:** 2021-08-30

**Authors:** Jacqui A. McGovern, Nathalie Bock, Abbas Shafiee, Laure C. Martine, Ferdinand Wagner, Jeremy G. Baldwin, Marietta Landgraf, Christoph A. Lahr, Christoph Meinert, Elizabeth D. Williams, Pamela M. Pollock, Jim Denham, Pamela J. Russell, Gail P. Risbridger, Judith A. Clements, Daniela Loessner, Boris M. Holzapfel, Dietmar W. Hutmacher

**Affiliations:** 1 Centre in Regenerative Medicine, Queensland University of Technology (QUT), Brisbane, QLD Australia; 2 School of Mechanical, Medical and Process Engineering (MMPE), Centre for Biomedical Technologies, Faculty of Engineering, QUT, Brisbane, QLD Australia; 3 School of Biomedical Sciences at Translational Research Institute (TRI), Faculty of Health, QUT, Brisbane, QLD Australia; 4 Australian Prostate Cancer Research Centre—Queensland (APCRC-Q), QUT, Brisbane, QLD Australia; 5 Queensland Bladder Cancer Initiative (QBCI), Brisbane, QLD Australia; 6 UQ Diamantina Institute, Translational Research Institute, The University of Queensland, Brisbane, QLD Australia; 7 Herston Biofabrication Institute, Metro North Hospital and Health Service, Brisbane, QLD Australia; 8 Musculoskeletal University Centre Munich, Department of Orthopedics and Trauma Surgery, University Hospital Munich, Ludwig-Maximilians University, Campus Großhadern, Munich, Germany; 9 Department of Pediatric Surgery, Dr. von Hauner Children’s Hospital, Ludwig-Maximilians-University of Munich, Munich, Germany; 10 School of Medicine and Population Health, University of Newcastle, Callaghan, NSW Australia; 11 Department of Anatomy and Developmental Biology, Faculty of Medicine, Nursing and Health Sciences, Monash University, Melbourne, VIC Australia; 12 Department of Chemical Engineering and Department of Materials Science and Engineering, Faculty of Engineering, Monash University, Melbourne, VIC Australia; 13 ARC Industrial Transformation Training Centre in Additive Biomanufacturing, QUT, Brisbane, QLD Australia

**Keywords:** Tissue engineering, Cancer models

## Abstract

Prostate cancer (PCa) is the second most commonly diagnosed cancer in men, and bone is the most frequent site of metastasis. The tumor microenvironment (TME) impacts tumor growth and metastasis, yet the role of the TME in PCa metastasis to bone is not fully understood. We used a tissue-engineered xenograft approach in NOD-*scid* IL2Rγ^null^ (NSG) mice to incorporate two levels of humanization; the primary tumor and TME, and the secondary metastatic bone organ. Bioluminescent imaging, histology, and immunohistochemistry were used to study metastasis of human PC-3 and LNCaP PCa cells from the prostate to tissue-engineered bone. Here we show pre-seeding scaffolds with human osteoblasts increases the human cellular and extracellular matrix content of bone constructs, compared to unseeded scaffolds. The humanized prostate TME showed a trend to decrease metastasis of PC-3 PCa cells to the tissue-engineered bone, but did not affect the metastatic potential of PCa cells to the endogenous murine bones or organs. On the other hand, the humanized TME enhanced LNCaP tumor growth and metastasis to humanized and murine bone. Together this demonstrates the importance of the TME in PCa bone tropism, although further investigations are needed to delineate specific roles of the TME components in this context.

## Abstract (web summary)

McGovern et al. establish an orthotopic, humanized prostate cancer model to study bone metastasis. The authors demonstrate that a humanized tumor microenvironment (TME) influences the metastatic spread of cancer cells to tissue-engineered bone, highlighting the importance of the TME in prostate cancer metastasis to bone.

## Introduction

Prostate cancer (PCa) is the second most commonly diagnosed cancer of men in the Western world^1^. While the localized disease can be treated with surgical excision and/or radiotherapy, thereby removing and controlling the primary tumor, the mortality of PCa typically is caused by the development of distant metastases, predominantly in the bone^2^. However, the metastatic features of preferential homing to the bone organ by PCa are not fully understood. In order to better comprehend these interactions, we have previously developed a humanized bone within a murine host using tissue-engineering techniques as a platform to study the interaction of human cancer cells with a human bone environment^3,4^. The human tissue-engineered bone construct (hTEBC) contains human bone cells and a human-derived extracellular matrix (ECM), and once implanted in vivo forms a humanized bone organ within the murine host^5^. We have used the hTEBC to investigate species-specific homing of PCa cells following intracardiac injection^3,6^. In this model, we showed that PCa cells preferentially grew in the hTEBC and were more responsive to zoledronic acid, a bisphosphonate that reduces osteoclast-mediated bone resorption, as compared to PCa cells which metastasized to the murine bone^6^. Furthermore, we demonstrated in a humanized, orthotopic xenograft model of PCa bone metastasis that the primary tumor microenvironment (TME) had the ability to influence the bone metastatic behavior of PCa cells^7^, confirming that the hTEBC is a valuable platform for studying PCa metastasis to human bone.

The local TME is an important regulator of primary tumor growth and metastatic spread to distant sites, such as the bone^8–11^. The TME consists not only of the malignant cells, but also many non-malignant cell types including fibroblasts, immune cells, vascular and lymphatic endothelial cells, as well as ECM components. Fibroblasts from the TME like cancer-associated fibroblasts (CAFs) and blood and lymphatic microvascular endothelial cells (MVECs) have been implicated in PCa metastasis, indicating their importance in the prostate TME^12–14^. CAFs isolated from PCa patients undergoing radical prostatectomy stimulated the tumorigenic growth of prostate epithelial cells, yet normal human prostate fibroblasts (NPFs) did not^15^. In an orthotopic mouse model, CAFs promoted PCa cell proliferation and tumor development compared to the PCa cells alone^16^. Nguyen et al. found that the CAF proteome was enriched for ECM regulatory proteins compared to NPFs in human PCa^17^. Additionally, in orthotopic PCa xenograft models, co-injection of human PCa cell lines with human MVECs resulted in enhanced metastasis to the lymph nodes^18^ and stimulated PCa cell autophagy thereby enhancing cellular metastatic invasive properties^12^. Previous findings from our laboratory using an orthotopic xenograft model demonstrated that co-injection of human PCa cells with human CAFs and blood and lymphatic MVECs resulted in decreased metastasis of PCa cells to hTEBCs when compared to tumors initiated from PCa cells alone^7^. Together these results emphasize the importance of CAFs and MVECs from the human TME in regulating PCa growth and metastasis.

Humanized xenograft models are vital for studying the role of the TME in PCa growth and subsequent metastasis to bone, as the development of spontaneous PCa in mice is rare^19^. The mouse prostate not only differs from the human prostate at an anatomical level, but also the stromal compartment of the human prostate is more abundant than in the murine prostate, which raises questions of the comparability between the two species^20,21^. Thus, from a clinical and therapeutic point of view, in order to effectively investigate the role of the TME on PCa growth and metastasis, orthotopic mouse models incorporating both malignant and non-malignant human cells from the tumor and the TME are pivotal^22^.

**Fig. 1:**
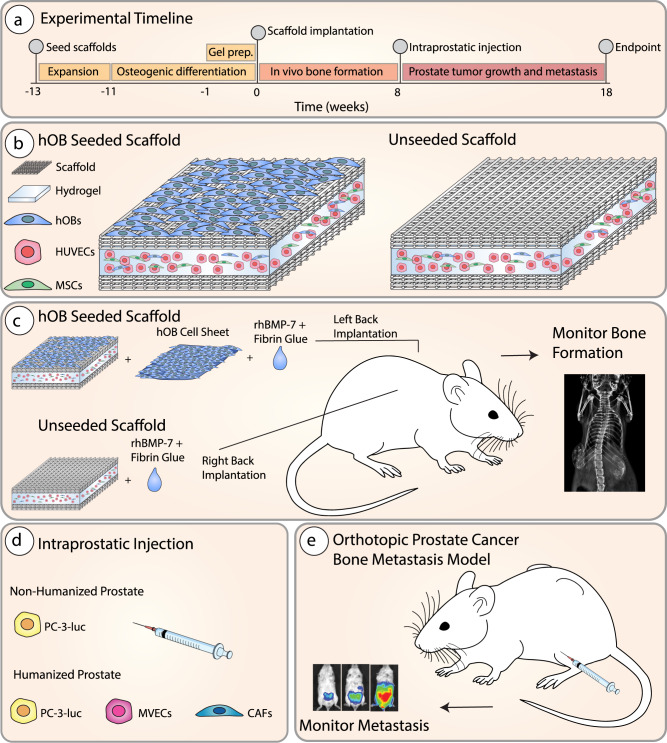
Schematic of experimental design. **a** Overview of the experimental timeline for the study. The human tissue-engineered bone construct (hTEBC) was created by seeding human osteoblasts (hOBs) onto quadratic calcium phosphate-coated melt electrowritten medical-grade polycaprolactone (mPCL) scaffold sheets with 95% porosity. The hOBs formed a dense cell/extracellular matrix (ECM) network throughout the scaffold architecture over 2 weeks before culture conditions were switched to osteogenic media for 11 weeks. **b** One week before implantation, star-shaped polyethylene glycol (sPEG) heparin gels were prepared containing hOBs, human mesenchymal stromal cells (MSCs), and human umbilical vein endothelial cells (HUVECs) and cultured to form capillary-like networks. A total of *n* = 10 mice were used for this study. Prior to subcutaneous implantation the sPEG-heparin gels were sandwiched together between two quadratic in vitro-engineered constructs. **c** The left-back of the male NSG mice received in vitro engineered constructs seeded with hOBs and combined with hOB cell sheets within the fibrin glue (in n-10 mice), whereas the right-back received unseeded scaffolds with the cell-loaded sPEG-heparin gel (in *n* = 10 mice). Both the cell-laden and cell-free in vitro engineered constructs were combined with recombinant human bone morphogenetic protein-7 (rhBMP-7) and fibrin glue immediately prior to implantation. The bone was allowed to develop for 8 weeks before intraprostatic injection and monitored using X-ray. **d** Humanization of the mouse prostate was performed via injection of PC-3-luc cells together with prostate lymphatic and blood vessel endothelial cells (BVECs) and PCa-derived fibroblasts (CAFs) performed in *n* = 5 mice, whereas the non-humanized group received an intraprostatic injection of PC-3-luc cells alone performed in *n* = 5 mice. **e** Growth of the primary prostate tumor and metastasis to distant organs were monitored weekly using in vivo bioluminescent imaging for 10 weeks. The mouse schematic image was sourced from Wikimedia Commons^52^.

We have previously observed that a humanized prostate TME decreased metastasis of PC-3 PCa cells to hTEBCs^7^. However, these PC-3 cells metastasized rapidly in vivo, therefore we hypothesized that using a less aggressive PC-3 variant, which permits slower prostate tumor growth and metastasis, in addition to the slower-growing LNCaP cell line, would allow us to observe a greater influence of the humanized prostate TME. Furthermore, we hypothesized that the presence of a humanized, bone-derived ECM was important for PCa metastasis to bone in hTEBCs. To investigate this, we generated bone organs of primarily human or murine origin in this study. This model was established on tissue-engineering principles and allows us to study bone metastasis of PCa cells primed by a human TME at the orthotopic tumor site to tissue-engineered bone constructs, encompassing two levels of humanization at both the primary and secondary sites (Fig. 1). We show that human PCa cells metastasized from the orthotopic tumor to the tissue-engineered bone in all mice, and importantly that a humanized TME appeared to decrease the metastatic spread of PC-3 PCa cells to tissue-engineered bone, but did not affect PCa metastasis to the endogenous murine bones or other organs. Although the humanized TME increased primary LNCaP tumor growth and metastatic spread to the humanized and murine bones, most endogenous murine organs did not present a significantly higher secondary tumor burden.

## Results

### Pre-cultured and highly mineralized hTEBCs formed a humanized bone environment in vivo

**Fig. 2:**
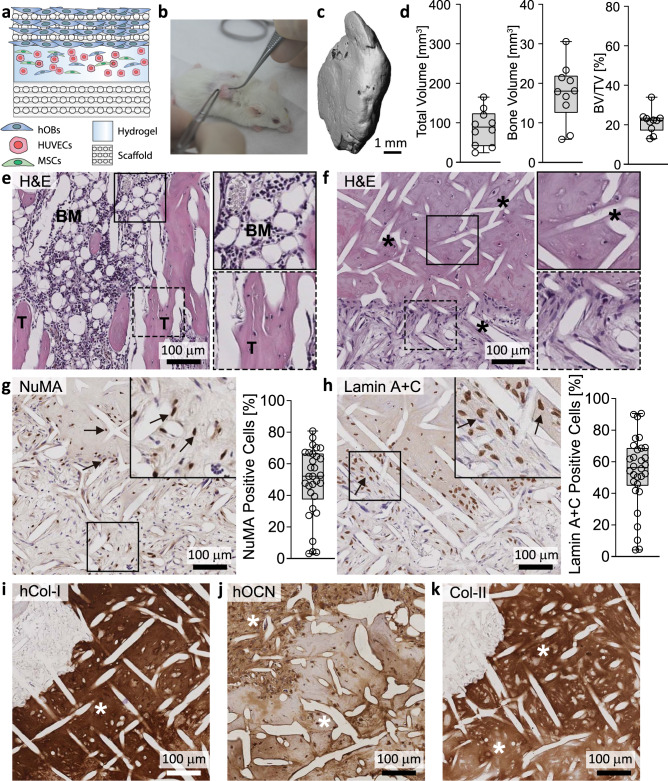
Sandwich scaffolds infiltrated with hOB-derived dense mineralized matrix form a humanized bone environment in vivo. **a** sPEG-heparin gels seeded with hOBs, MSCs, and HUVECs were sandwiched between two mPCL-CaP scaffolds and **b** subcutaneously implanted into the back of NSG mice together with fibrin glue and rhBMP-7 to stimulate bone formation. **c** Ex vivo micro-CT reconstruction shows calcified tissue formation after in vivo implantation of the hTEBCs and **d** bone volume (mm^3^), total volume (mm^3^), and bone volume to total volume ratios (%) are similar to previously published data (*n* = 10 hOB seeded hTEBC)^3^. **e** Histological analysis of the hOB seeded hTEBC shows that the scaffold formed into a functional organ bone containing marrow (BM; upper inset panel), interspersed with calcified trabecula bone tissue (T; lower inset panel). **f** Calcified bone tissue was generated by the hOBs seeded on the scaffolds and formed around the mPCL-CaP fibers (upper inset panel; indicated by the black asterisks), cells in uncalcified tissue areas are still found adjacent to the bone (lower inset panel). **g** Antibodies raised against NuMA and **h** Lamin A + C (brown immunoreactivity, indicated by the black arrows) show that the hOBs were incorporated into the chimeric organ bone, which is made of human and mouse tissue. Images were captured from three sites of the tissue section from each of the *n* = 10 hOB seeded hTEBCs and the percentage of positive cells was quantified (*n* = 30 images from 10 biological replicates). **i** Bone matrix proteins type I collagen (hCol-I) and **j** osteocalcin (hOCN) were detected using antibodies that react with human but not murine tissues, demonstrating that bone matrix proteins were produced by the hOBs (indicated by the white asterisks). **k** The presence of type II collagen (Col-II) shows that the calcified bone tissue was produced via endochondral ossification (indicated by the white asterisks). The scale bars represent 100 µm. Data are represented as box plots depicting the median, first and third quartile, minimum and maximum, and are overlaid with individual data points.

The hTEBC was created using CaP-coated mPCL scaffolds (Supplementary Fig. S1a–c), which were seeded with human osteoblasts (hOBs; Supplementary Fig. S1d) and cultured under osteogenic conditions to form a highly mineralized bone matrix (Fig. 1a–c and Supplementary Fig. S1e, f). The presence of the hOB cell layer and cell viability was confirmed using live/dead staining and confocal laser scanning microscopy in parallel samples prior to implantation in vivo (Supplementary Fig. S1g, h). In order to pre-vascularize the hTEBC, a star-shaped polyethylene glycol (sPEG)-heparin hydrogel pre-cultured with human umbilical vein endothelial cells (HUVECs)^23^ and a supportive population of human placental mesenchymal stromal cells (MSCs) and hOBs was sandwiched between two mPCL-CaP scaffolds (Figs. 1b–c and 2a). The left-back of the mice received mPCL-CaP scaffolds seeded with hOBs, whereas the right received unseeded mPCL-CaP scaffolds (Figs. 1b, c and  2a, b). Both mPCL-CaP scaffold groups (hOB seeded and unseeded) contained the pre-vascularized sPEG-heparin hydrogels. The rationale for this was to generate an ectopic bone of human origin that contained human-derived bone matrix components and compare this to a bone organ of largely murine origin in order to investigate if preferential metastasis of human PCa cells to humanized bone occurred. There were no significant differences in the total volume (TV, mm^3^) of the implanted bone constructs between the hOB seeded and unseeded groups (*P* = 0.659) (Supplementary Fig. S2c). This was expected since the implanted constructs were the same size. Interestingly, there were also no significant differences in bone volume (BV, mm^3^; *P* = 0.458) or bone volume fraction (BV/TV, %; *P* = 0.880) in mice whose bone organ was comprised predominantly of human cells, as compared to those whose bone organ was comprised primarily of host-derived murine cells (Supplementary Fig. S2c). The combined tissue-engineered bone construct data showed that the sandwich scaffolds formed a bone organ with a similar BV and BV/TV as previously published when using tubular scaffold constructs (Fig. 2c, d)^3,4^. Similarly, the implanted hTEBCs formed a bone organ containing trabeculae and bone marrow (Fig. 2e and Supplementary Fig. S2d) and ossified forming a cortical shell (Fig. 2f and Supplementary Fig. S2d). Human cells were observed throughout the hOB seeded scaffold architecture and inner cancellous bone of the hOB seeded hTEBC as indicated by antibodies that have been validated for their cross-reactivity with human but not murine tissues by immunohistochemistry (IHC)^5,7^. Nuclear mitotic apparatus protein 1 (NuMA), present in the cell nucleus (Fig. 2g), and Lamin A + C, expressed in the nuclear envelope (Fig. 2h), staining indicated that human cells accounted for approximately 50–60% of all cells present in the hOB seeded humanized bone organ in vivo. This indicated that the engrafted human cells persisted within the murine environment for approximately 4 months after implantation. The ECM composition of the hOB seeded hTEBC was further investigated using IHC. The presence of large quantities of human-derived type I collagen (hCol-I; Fig. 2i) confirmed that the hOBs produced a human-derived ECM within the murine host. Osteocalcin (hOCN), a protein produced solely by osteoblasts and the most definitive marker for bone formation, was expressed in the hTEBC as determined using an antibody that detects human, but not murine OCN (Fig. 2j). The presence of type II collagen (Col-II; Fig. 2k) was indicative that the bone had formed through endochondral ossification as opposed to direct calcification, which is more physiologically relevant for the process for marrow-containing bone formation^24^.

In order to determine the impact of hOB pre-culture and pre-mineralization on human ECM deposition in the implanted hTEBC, the left-back of the mice received a sandwich mPCL-CaP scaffold which had been seeded and pre-cultured with hOBs, whereas the right-back contained a sandwich mPCL-CaP scaffold in which there were no seeded hOBs (unseeded; Supplementary Fig. S2a). Both implanted hTEBCs formed a bone organ with cortical shell and bone marrow (Supplementary Fig. S2b). As expected, hOB-seeded scaffolds contained a significantly higher proportion of hCol-I than the unseeded scaffold group (*P* = 0.045, Supplementary Fig. S2e, f), as well as a significantly higher percentage of NuMA- and Lamin A + C-positive cells compared to the unseeded scaffold group, (*P* = 0.000037 and *P* = 0.000834, respectively; Supplementary Fig. S2e, f). The presence of human cells and deposition of ECM components in tissue-engineered bone from the unseeded group suggested that the MSCs and hOBs present in the sPEG-heparin construct migrated from the hydrogel, populated the unseeded mPCL-CaP scaffold, and assisted in the cortical bone formation. However, the level of human cells and ECM was significantly greater when pre-seeding with hOBs was performed. Together this data suggests that pre-seeding mPCL-CaP scaffolds with hOBs are not required for ectopic organ bone formation, but hOB pre-seeding does promote the generation of a robust humanized bone microenvironment with increased deposition of human ECM.

### Orthotopic and humanized prostate tumors were induced by intraprostatic injection of PCa cells

**Fig. 3:**
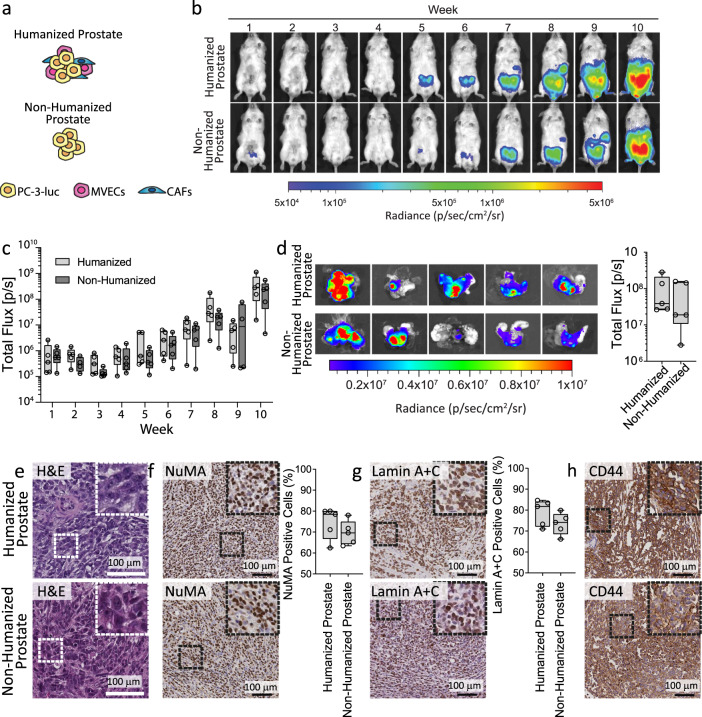
Primary orthotopic prostate tumor development and characterization. **a** Intraprostatic injection of the human prostate cells was performed into the dorsal lobe of the murine prostate. **b** Representative whole body in vivo bioluminescent images (BLI) over the 10 weeks following intraprostatic injection. **c** Quantification of in vivo BLI signal in mice with the humanized (light gray box plots) compared to the non-humanized (dark gray box plots) prostate microenvironment (*n* = 5 mice per group). **d** Ex vivo BLI images of murine prostates and correlating quantification of ex vivo BLI signal from the mouse prostates at the experimental endpoint (*n* = 5 prostates per group) reveals that there are no significant differences between the groups using a Mann−Whitney U Test. **e** Hematoxylin and eosin (H&E) staining of the primary tumor. Immunohistochemistry staining for (**f**) NuMA and (**g**) Lamin A + C using antibodies that react with human but not murine tissues was performed. Images were captured from three sites of the tissue section, and the percentage of human cells relative to total cells was quantified and averaged per prostate sample (*n* = 5). Data were analyzed using an unpaired *t*-test and no significant differences were found. **h** CD44 immunohistochemistry was performed as an additional marker for human cells. The scale bars represent 100 µm. Data represented in box plots depict the median, first and third quartile, minimum and maximum, and is overlaid with individual data points.

**Fig. 4:**
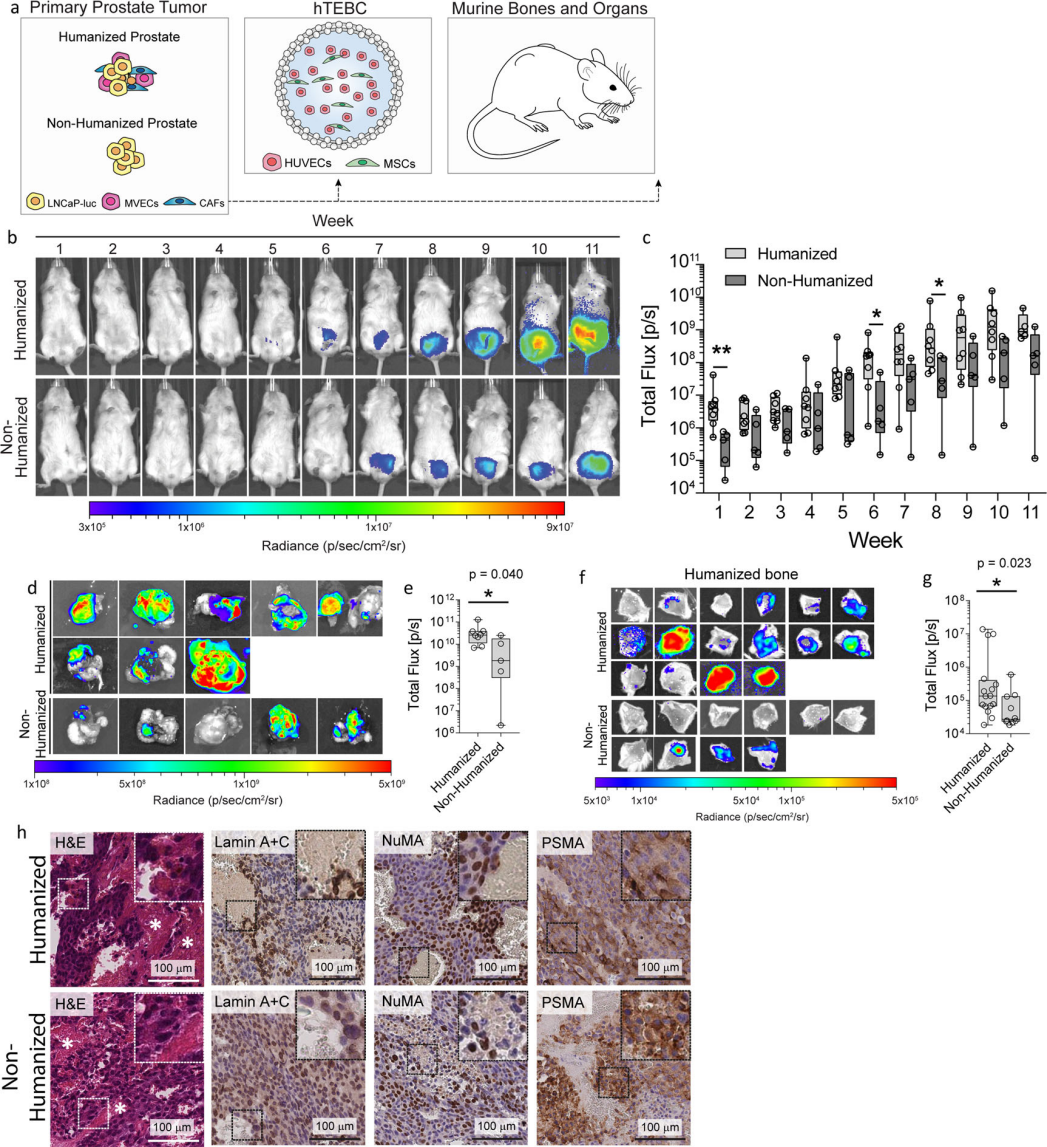
Growth of LNCaP-luc primary prostate tumors and metastasis from the primary prostate tumor to the hTEBC. **a** Overview of the different levels of humanization within the in vivo model. The prostate was humanized by co-injection of LNCaP-luc, BVECs, and CAFs into the murine prostate. The back of the mice received humanized bone constructs with hOB seeded scaffolds. **b** Representative whole body in vivo bioluminescent images (BLI) over the 11 weeks following intraprostatic injection. **c** Quantification of in vivo BLI signal in mice with the humanized (light gray box plots) compared to the non-humanized (dark gray box plots) prostate microenvironment (*n* = 5 mice in the non-humanized prostate group and *n* = 8 mice in the humanized prostate group). **d** Ex vivo BLI images of murine prostates and **e** correlating quantification of ex vivo BLI signal from the mouse prostates at the experimental endpoint (*n* = 5 in the non-humanized prostate group and *n* = 8 prostates in the humanized prostate group) reveals that there was a significant difference between the groups. **f** Representative ex vivo BLI of the hTEBCs with LNCaP-luc PCa metastases and **h** quantification of the BLI data from LNCaP-luc cells which had metastasized from the primary prostate tumor to the hTEBC (*n* = 10 scaffolds in the non-humanized prostate group and *n* = 16 scaffolds in the humanized prostate group) reveals increased LNCaP metastasis from the humanized prostate to the hTEBC. **g** Hematoxylin and eosin (H&E) staining of the primary tumor. Immunohistochemistry staining for NuMA and Lamin A + C using antibodies that react with human but not murine tissues were performed. Prostate-specific membrane antigen (PSMA) immunohistochemistry was performed as an additional marker for human LNCaP cells. The scale bars represent 100 µm. Data are represented as box plots depicting the median, first and third quartile, minimum and maximum, and are overlaid with individual data points. All data were not normally distributed and were analyzed using a Mann−Whitney U test. The mouse schematic image was sourced from Wikimedia Commons^52^.

In our previous work, we generated an orthotopic and humanized tumor within the murine prostate using PC-3 cells stably expressing luciferase (PC-3-luc)^7^. We reported that prostate humanization with fibroblasts from human prostate tumor samples (CAFs) and primary human prostate CD31 + blood and lymphatic MVECs reduced metastasis of PC-3-luc cells to the hTEBC. However, the PC-3-luc cells used were aggressive, metastasizing quickly, and the experiment was terminated 4 weeks after PC-3-luc injection. Thus, we hypothesized that using a less aggressive variant of the PC-3 PCa cell line and the slower growing LNCaP cell line, would allow slower prostate tumor growth and metastasis, and would enable us to observe a greater influence of the humanized prostate microenvironment. The growth characteristics of the previously used PC-3-luc (PC-3-luc1) cells^7^ and the same PC-3-luc cell line from a different laboratory (PC-3-luc2) were compared to parental PC-3 cells which did not express luciferase and are routinely used in our laboratory. The previously used PC-3-luc1 cell line^7^ proliferated more quickly in 2D and 3D as compared to the PC-3-luc2 and parental PC-3 cells, but all of the PC-3 cell lines were morphologically similar (Supplementary Fig. S3). Therefore, to establish a humanized orthotopic prostate environment in our xenograft approach, MVECs and CAFs were injected into the prostate of immunocompromised NOD-*scid* IL2Rg^null^ (NSG) mice together with the less aggressively growing, osteotrophic PC-3-luc PCa cells (PC-3-luc2), which will be referred to as ‘PC-3-luc’ hereafter. In another cohort, LNCaP-luc cells were injected into the murine prostate together with the MVEC and CAF cells. Mice receiving only PC-3-luc or LNCaP-luc cells served as a non-humanized prostate control (Figs. 1d,  3a, and 4a). Tumor growth in the murine prostate was monitored over the duration of the experiment using in vivo bioluminescence imaging (BLI) to detect the signal from the luciferase-expressing PC-3luc (Fig. 3b) or LNCaP-luc (Fig. 4b) PCa cells. The luciferase activity increased in a progressive manner, with no significant differences in PC-3 BLI signals between the humanized and non-humanized prostate tumor groups (Fig. 3c). A significantly higher BLI signal was observed in the LNCaP-luc humanized prostate group compared to the non-humanized LNCaP prostate group at weeks 1 (*P* = 0.008), 6 (*P* = 0.028), and 8 (**P = 0.04) (Fig. 4c). Ex vivo BLI analysis of the prostate tissues at the endpoint confirmed that there were no significant differences in tumor burden between the PC-3 humanized prostate tumors compared to the non-humanized prostate tumors (*P* = 0.347), but a significantly greater tumor burned in the humanized LNCaP prostate compared to the non-humanized LNCaP prostate was observed (*P* = 0.040). Together, this suggests that humanization of the prostate stromal component did not influence the growth of the primary PC-3 prostate tumor, but did influence the growth of the primary LNCaP tumor (Figs. 3d,  4d, e and Supplementary Table S1). Intraprostatic injection of PC-3-luc cells resulted in a prostate tumor morphologically representing poorly differentiated prostatic carcinoma with prominent nucleoli (Fig. 3e). While the LNCaP-luc primary tumors in both groups featured areas of necrosis and blood lakes^9^ (Fig. 4f). The orthotopic prostate tumors were probed with antibodies against NuMA (Figs. 3f and 4f), Lamin A + C (Figs. 3g and 4f), and CD44 (Fig. 3h) or prostate-specific membrane antigen (PSMA; Fig. 4f), to distinguish between cells of human and murine origin. The relative percentage of human cells in the prostate tissues was quantified to determine if the inclusion of human fibroblasts and MVECs influenced the total proportion of human cells present within the murine prostate. The percentage of NuMA and Lamin A + C positive cells did not differ between the humanized and non-humanized prostate environments (*P* = 0.18 and *P* = 0.298, respectively), thus we concluded that the addition of fibroblasts and ECs did not influence the overall human cell density within the murine prostate in our xenograft approach. Furthermore, hCol-I, a key ECM protein produced by fibroblasts, was not detected in either the humanized or non-humanized prostate environments (Supplementary Fig. S4). Together, these data confirm that humanization of the murine prostate environment with human CAFs and MVECs did not enhance primary tumor growth of PC-3-luc cells, nevertheless did enhance the growth of LNCaP-luc cells in vivo.

### A humanized prostate microenvironment decreased the metastasis of PC-3-luc cells to tissue-engineered human bone

**Fig. 5:**
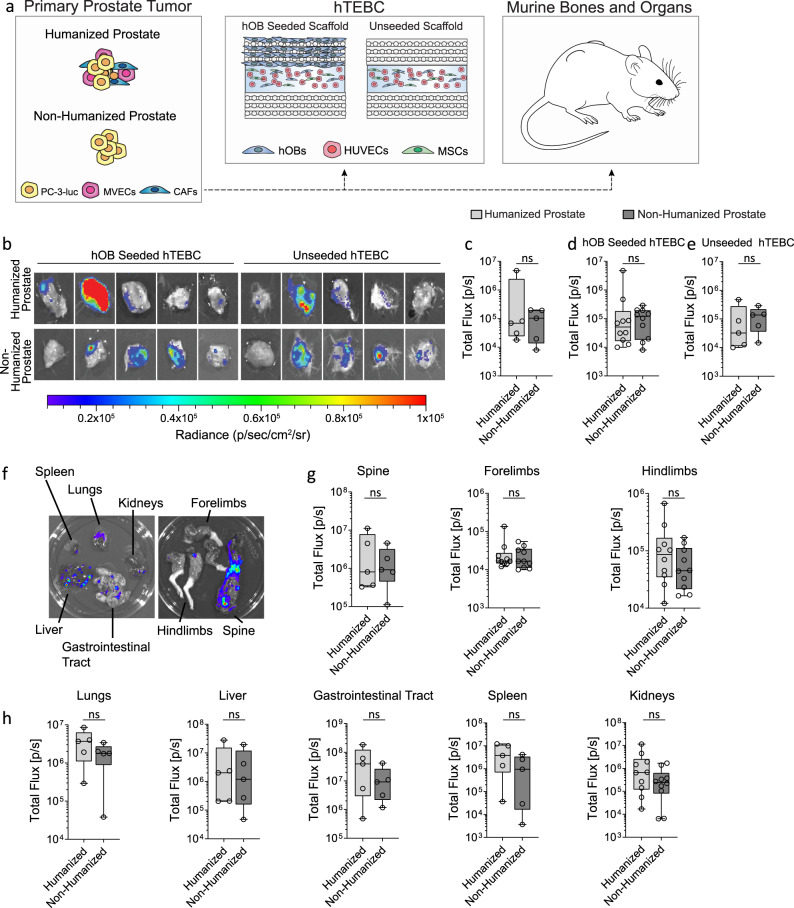
Metastasis of PC-3-luc cells from the primary prostate tumor to the hTEBC, murine bones, and organs. **a** Overview of the different levels of humanization within the in vivo model. The prostate was humanized by co-injection of PC-3-luc, BVECs, and CAFs into the murine prostate. The back of the mice received humanized bone constructs with scaffolds seeded or unseeded with hOBs. The murine bones and organs, in addition to the hTEBCs were analyzed for PC-3-luc metastases. **b** Representative ex vivo bioluminescent images (BLI) of the hTEBCs with PC-3-luc PCa metastases and **c** quantification of the BLI data from PC-3-luc cells which had metastasized from the primary prostate tumor to all hTEBC (data from hOB seeded and unseeded hTEBC combined, *n* = 10 hTEBC from five mice with two scaffolds each). **d** Quantified BLI data were grouped based on metastasis of PC-3-luc cells to the hOB-seeded scaffold (left-back only; *n* = 5 hTEBCs from five mice), or **e** the unseeded scaffold (right-back only; *n* = 5 hTEBCs from five mice). **f** PCa metastases were present in murine organs and bones as indicated by ex vivo BLI. **g** No significant differences in metastasis from the humanized or non-humanized prostate tumors to the murine bones; spine, forelimbs, or hindlimbs. **h** No significant differences in metastasis to the murine organs; lungs, liver, gastrointestinal tract, spleen, or kidneys. Data are represented as box plots depicting the median, first and third quartile, minimum and maximum, and are overlaid with individual data points. All hTEBC, lung, and spleen BLI data were normally distributed and analyzed using an unpaired *t*-test. The hOB seeded and unseeded hTEBC, spine, forelimb, hindlimb, liver, gastrointestinal tract, and kidney BLI data were not normally distributed and were analyzed using a Mann−Whitney test. The mouse schematic image was sourced from Wikimedia Commons^52^.

The metastatic burden of PC-3-luc cells from the humanized and non-humanized prostate to the hTEBC and distant murine bones and organs (Fig. 5a) was examined using ex vivo BLI (Fig. 5b–h). The ex vivo BLI data was quantified as an indicator of the metastatic PCa burden in the hTEBC (Fig. 5b–e and Supplementary Table S2), the murine bones (Fig. 5f, g and Table S3), and the other murine organs (Fig. 4h and Supplementary Table S3). We have previously observed that the humanized prostate TME reduced metastasis of PC-3-luc cells to tissue-engineered bone. We observed a similar trend in this study when we combined the data from the hOB seeded and unseeded hTEBC BLI data, although this was not statistically significant (*P* = 0.545) (Fig. 4c)^7^. Furthermore, if the BLI data was separated, the presence of a humanized bone microenvironment (hOB seeded hTEBC; *P* = 0.917) was no more favorable as a metastatic niche for PC-3-luc cells than an ectopic bone organ comprised predominantly of murine ECM (unseeded hTEBC; *P* = 0.347) (Fig. 4d, e). Although, the trend suggests that metastatic burden from the humanized prostate to both the hOB seeded and unseeded hTEBC was lower compared to the non-humanized prostate. Humanization of the prostate TME did not influence the frequency or metastatic burden of PC-3-luc cells to the murine bones (spine; *P* = 0.917, forelimbs; *P* = 0.821, and hindlimbs; *P* = 0.345) (Fig. 4f, g), lungs (*P* = 0.329), liver (*P* = 0.917), gastrointestinal tract (P = 0.465), spleen (*P* = 0.183) or kidneys (*P* = 0.199) (Fig. 4h). Together, this data suggests that tissue engineering a humanized prostate niche within a mouse model does not significantly influence PC-3 PCa cell metastasis to tissue-engineered bone, murine bones, or other organs in this model. Additionally, ectopic bone comprised primarily of human ECM as opposed to primarily endogenous murine ECM did not influence the metastatic homing of human PCa cells in vivo. As human ECM was present in both seeded and unseeded ectopic bone environments, we cannot conclude at this stage if species-specific ECM is a driver for metastatic homing of PCa cells in this model.

### Bone metastatic capabilities of LNCaP-luc PCa cells was enhanced by a humanized prostate microenvironment

Metastasis of LNCaP-luc cells from the humanized and non-humanized prostate to the hTEBC (Fig. 4g) and distant murine bones and organs was examined using ex vivo BLI. The ex vivo BLI data was quantified as an indicator of the metastatic PCa burden in the hTEBC (Fig. 4h and Supplementary Table S4), the murine bones, and the murine visceral organs (Supplementary Fig. 4 and Supplementary Table S5). Here, we observed a statistically significant increase in PCa metastatic burden in the hTEBC when LNCaP-luc metastasis occurred from the humanized prostate TME compared to the non-humanized prostate TME (*P* = 0.023) (Fig. 4g). Interestingly, humanization of the LNCaP-luc prostate TME also resulted in significantly greater tumor burden in the murine spine (*P* = 0.040), forelimbs (*P* = 0.000096), and hindlimbs (*P* = 0.004) when compared to the non-humanized LNCaP-luc prostate TME (Supplementary Fig. S4). Analysis of ex vivo BLI data of the murine visceral organs revealed that humanization of the prostate TME did not influence the frequency or metastatic burden of LNCaP-luc cells in the lungs (*P* = 0.143), liver (*P* = 0.143), gastrointestinal tract (*P* = 0.143), or spleen (*P* = 0.079), but did increase the metastatic burden of LNCaP-luc cells in the kidneys (*P* = 0.006) (Supplementary Fig. S5). Together, this data suggests that a humanized, tissue-engineered LNCaP prostate TME enhances the metastatic colonization of PCa cells to human and murine bone microenvironments, although it does not influence LNCaP metastatic spread to the murine visceral organs.

## Discussion

Advanced PCa predominantly metastasizes to bone, which is the incurable stage of the disease^1,2^. To better understand the mechanisms behind PCa bone metastasis, and to develop more effective treatment strategies, improved in vivo models which more accurately reflect PCa disease progression are necessary^22^. However, this undertaking is limited by animal models in which PCa metastasis pathophysiology is not representative of the human condition. A quest for enhanced humanization in animal models has led to a number of novel humanized models from our group and others in recent years. These advanced models have specifically demonstrated differences in response to either metastasis rates, location of metastases, or response to treatments^3–7,25^. However, it is still unclear whether the humanization of both the primary TME and secondary metastatic organs provides a significant advantage. We sought to address this question with the partial humanization of both the primary organ (prostate) and the receiving organ (bone tissue) within a murine system. Specifically, in this study, an orthotopic and humanized prostate tumor was generated to study species-specific metastasis of PC-3 and LNCaP PCa cells to a humanized tissue-engineered bone environment (hTEBC). We hypothesized that the humanization of the murine prostate tissue with MVECs and CAFs primes PCa cells to metastasize to the hTEBC in a species-specific manner. We observed that humanization through co-injection of CAFs and MVECs into the murine prostate did not enhance primary tumor growth of PC-3 cells, yet did enhance the growth of an LNCaP primary tumor. In line with our previous findings^7^, we did observe that a humanized prostate TME appeared to reduce the metastasis of PC-3 cells to tissue-engineered bone, but did not influence metastasis to the murine bones or organs. Furthermore, and in support of our hypothesis, we observed that a humanized prostate TME enhanced LNCaP cell metastasis to the bone microenvironment.

We have previously shown that PC-3 cells exhibit the decreased metastatic potential to the humanized bone when grown in a primary tumor containing a humanized TME consisting of CAFs and MVECs^7^. The rapid and aggressive growth of the PC-3 cells may have influenced metastatic spread to the bone and other organs, therefore we hypothesized that using a slower growing PC-3 cell line, whose growth is consistent with the original parental PC-3 cells in our laboratory, may allow us to decipher the impact of the prostate TME on cancer-to-bone metastatic behavior.

In line with our finding of the different phenotypic growth characteristics of the PC-3 cells, genetic diversification, phenotypic and functional variations of other commonly used cell lines have been reported. In one study, the metastatic MCF-7 breast cancer cell line was sampled from different laboratories and subjected to comprehensive analyses. The sub-strains of MCF-7 cells were shown to have widely altered responses to anti-cancer drugs, with some being inhibited by certain anti-cancer compounds whereas others were unaffected^26^. Furthermore, large differences in MCF-7 cell morphology and proliferation in response to estradiol were observed between laboratories, despite the MCF-7 cell line identity being confirmed by short tandem repeat profiling. These differences were a result of genetic heterogeneity, which was traced back to the original stock vial purchased from the ATCC, indicating the inherently heterogeneous nature of cell lines^27^. In another study, the HEK239 cell line was reported to be non-tumorigenic in vivo at low passage number, but highly tumorigenic at high passage number, suggesting that non-malignant cell lines can undergo malignant transformation with prolonged culture time on tissue culture plastic^28^. Additionally, Liu et al. performed analysis on HeLa cell line samples from 13 different laboratories and found large variations in gene copy number and their proteomic profile. Consequently, there were also considerable differences in the HeLa sub-strain phenotype and population doubling times under standardized culture conditions^29^. Together, these studies suggest that the handling of a cell line in vitro, in addition to a cell line’s inherent genomic instability and the overall passage number has a large influence on the growth and overall behavior of a particular cell line.

Although cell lines have historically been considered a reproducible model system for cancer research, there is mounting evidence for cell line genomic instability and heterogeneity in growth characteristics and response to drug treatment^26,28^. The inherent limitations of using cell lines for PCa research need to be considered in future studies and may be overcome by using multiple different cell lines with similar invasive or metastatic behavior to confirm biological effects. Furthermore, when considering the effect of the TME on PCa tumor growth and metastasis, one must also consider the multicellular nature of the TME. The PCa TME consists of not only CAFs and MVECs, but also immune cell populations such as T lymphocyte subsets and tumor-associated macrophages (TAMs). The density of TAMs within PCa specimens has been associated with patient response to androgen deprivation therapy, decreased survival time, and overall poor prognosis^30^. Similarly, regulatory T cells (Tregs) are reported to be increased in PCa versus normal prostate tissue and are associated with suppression of anti-tumor responses from effector T cells^30^. Mauffrey et al. reported that neural progenitor cells can infiltrate the PCa TME of an orthotopic PC-3 xenograft model and establish new adrenergic neurons, which could sustain the development of PCa metastases^31^. A limitation of this study is the simplified humanized PCa TME, which consisted of only PCa cells, CAFs and MVECs. Humanizing the murine immune system through the introduction of human hematopoietic stems cells would also further humanize this model and account for the important role that TAMs and other immune cell populations play in cancer progression^25^. Furthermore, implementing patient-derived xenograft (PDX) models may be advantageous for future work too^32^. Primary PDX samples indeed include several TME components such as CAFs, MVECs, and immune cells, in addition to the malignant cells, and retain the individual characteristics from each patient. However, human stroma is gradually replaced with murine stroma with subsequent PDX passaging^33^.

The cellular prostate environment was humanized following intraprostatic co-injection of CAFs and MVECs, together with PC-3-luc or LNCaP-luc cells. We hypothesized that a less aggressively growing PC-3 cell line better interacts with this local TME promoting primary tumor growth and metastasis to bone. However, BLI revealed that there was no significant difference in the growth of the primary tumor between the humanized or non-humanized prostate groups. Histological analysis of the primary tumors revealed no obvious morphological or phenotypical differences between both groups and no differences in the level of human cellularity in the prostate tumor. This finding is in agreement with our previously published data^7^. Similarly, the inherently less aggressive LNCaP cell line was exploited to study the effects of the local TME on supporting the metastatic spread of the PCa cells. Here we did observe an increase in primary tumor growth and metastatic spread to the humanized and murine bones but did not see phenotypic or morphological differences in the primary tumor. The implanted TME cells may be unable to survive in vivo for the timeframe of this study. Fabris et al reported that injected CAFs persist for only 13 days in a murine subcutaneous mammary tumor model^34^. In an orthotopic mammary xenograft model, CAFs were replaced by host murine stromal cells within 3 weeks, but the transplanted endothelial cells persisted for up to 8 weeks in vivo^35^. Furthermore, Ippolito et al. subcutaneously injected PC-3 cells and CAFs in an immunocompromised mouse model. The authors observed evidence of CAF and PC-3 interaction via mitochondrial transfer but disappointingly did not observe the CAFs in the tumor at the endpoint 25 days after injection^36^. In an orthotopic and humanized PCa study, CAFs were co-injected together with LNCaP or LuCaP136 cells into the murine prostate. Linxweiler et al. observed increased metastases from the humanized prostate, but could not observe CAFs in histology sections of the primary tumor at the 10 week experimental endpoint^37^. Importantly, the LNCaP or LuCaP136 cells were injected in Matrigel, ECM derived from a murine tumor, which may have influenced the metastatic capability of the PCa cells they used^37^. A time-course study to investigate the cellular composition of the prostate at the histological level may inform us how long the stromal cells survive in our approach.

Although the TME has been implicated in PCa progression, the specific roles of the various cellular components of the prostate TME in PCa development and metastasis are not fully understood. The MVECs used here were isolated from a radical prostatectomy specimen. Zeng et al. reported that PCa cell lines (PC-3 and LNCaP) activated the MVECs, potentially through a VEGFR-2 dependent mechanism, and this can lead to the development of lymph node metastases^14^. Endothelial cells secrete CCL5, which downregulates androgen receptor expression and increases PCa cell invasion^12^. CCL5 is also produced by cells from the bone microenvironment and promotes PCa metastasis to bone^38^. The role of fibroblasts in the TME and their influence on tumor growth and metastasis is debated. Normal human fibroblasts enhanced the orthotopic engraftment of mammary epithelial cells in a mouse model of mammary tissue growth^39^. In contrast, human fibroblasts inhibited the orthotopic growth of malignant breast tissue in a mouse xenograft study^40^. In a PCa xenograft model, the influence of prostate stromal cells varied from patient to patient. In some cases, the patient-derived stromal cells enhanced tumor formation and increased host vascular recruitment to the tumor, whereas stromal cells from other patients had no impact on tumor formation compared to PCa cells alone^9^. Moreover, CAFs may play a role in prostate tumor development and metastasis mediating the deposition of human ECM and secretion of growth factors and proteases that support cancer cell proliferation and migration^41^. Nguyen et al. reported that CAFs differ from normal prostate fibroblasts (NPFs) at the proteomic level, and can enhance the proliferation of benign prostate epithelial cells, whereas NPFs do not possess this capability^17,42^. Interactions with the TME are important in tumor formation, yet the specific stromal factors which enhance tumorigenesis are complex.

Here we reported that humanization of the prostate TME enhanced the metastatic homing of LNCaP cells to the human and murine bones, while largely not influencing metastatic spread to the visceral organs. In contrast, the humanization of the prostate TME did not significantly influence the growth or metastasis of PC-3 cells in our model. Interestingly, the humanized PCa TME increased the bone tropism of the lymph node-derived LNCaP cell line but not the intrinsically bone metastatic PC-3 cell line. Chen et al. reported that CAFs increase the expression of cadherin-11, also known as osteoblast-cadherin, on MCF-7 and MDA-MB-231 breast cancer cells, which subsequently increased primary tumor growth and metastatic capabilities in vivo^43^. PC-3 but not LNCaP cells endogenously express cadherin-11, and reducing cadherin-11 expression in PC-3 cells reduced the incidence of bone metastases in an in vivo model^44^. The CAF-induced changes in cadherin-11 expression in LNCaP cells have not yet be established. Theoretically, CAFs present within the humanized TME may increase the expression of cadherin-11 or other proteins, which confer bone metastatic ability, thereby specifically influencing the bone metastatic capabilities of the LNCaP but not PC-3 PCa cells. Future studies to delineate the possible bone tropic priming events imparted by the TME on PCa cells may offer novel mechanistic insights into PCa metastasis to bone.

In our study, while we observed that a humanized prostate TME influenced the metastasis of PCa cells to the hTEBC, the identification of mechanisms responsible for this effect was not the purpose of this study. One limitation of our study was the design of the unseeded scaffold groups of the PC-3 study; ectopic bone in this group is not completely of murine origin. The presence of the MSCs and hOBs in the sPEG-heparin hydrogels served as a reservoir of human cells with osteogenic capacity that contributed to unexpectedly high bone formation and ECM deposition in the unseeded hTEBC group. Therefore, we cannot fully exclude that PCa metastasis to the hTEBC is specific to the humanized environment in the hOB seeded scaffold group as the unseeded scaffolds also contained human ECM, albeit to a significantly lesser extent.

The hTEBC forms a viable bone organ when implanted in vivo, and includes a cortical shell, trabecular bone, hematopoietic and fatty bone marrow, vasculature, as well as bone-resident osteoblasts, osteocytes, and osteoclasts^5^. Pre-culturing hOBs on mPCL-CaP scaffolds in vitro stimulates the production of the human-derived ECM within the construct, which leads to a bone organ of largely human origin^5^ which continues to be deposited and remodeled following in vivo implantation^45^. We have previously demonstrated that TEBC of human origin significantly differs from TEBC of murine origin^45^. Indeed, murine TEBC displayed similar canalicular density and parallel collagen fiber orientation as observed in native murine bone, in contrast to human TEBC which possessed a sparse canicular density and an interwoven collagen fiber alignment^45^. Here we observed that the MSCs and hOBs migrated from the co-implanted sPEG-heparin hydrogel and contributed to bone formation and ECM deposition in the unseeded scaffolds. However, the humanized ECM and cellular content were significantly lower than in the hOB seeded scaffolds, suggesting that the degree of humanization within tissue-engineered constructs is less related to the biomaterial construct chosen (scaffold versus hydrogel) but can be more stringently controlled via the numbers of human cells injected.

The hTEBC has been extensively reported to operate as a niche for cancer cell growth and metastasis in vivo^3,4,7,25^. We describe here that an hTEBC of mainly human origin (hOB seeded) and of mainly murine origin (unseeded) both serve as a metastatic homing site for PCa cells. It is unclear if the PCa cells metastasized to the hTEBC due to the presence of a humanized ECM, or if there are other factors from the hTEBC which attracted the metastatic PCa cells. Carpenter et al. suggested that implantable niches were a transiently pro-inflammatory microenvironment that can stimulate the homing of cancer cells from a primary tumor site^46^. Wound healing and localized inflammation from implanting the scaffolds were shown to influence the homing of cancer cells to the humanized tissue-engineered construct. Rao et al. found that implanted PCL scaffolds had a dynamic immune cell response that stabilized one month after implantation^47^. During this timeframe, the implanted scaffold served as a metastatic niche for breast cancer cells from a primary orthotopic tumor. Mice bearing an implanted niche had significantly reduced metastases in other organs; the implanted niche acted as a reservoir to trap metastatic cancer cells and prevented metastasis to other murine organs^47^. However, in the present study, we used immunocompromised mice, and there was no evidence of inflammation at the hTEBC site. PC-3 and LNCaP cells were introduced 7−8 weeks after scaffold implantation, thereby leaving enough time for any transient inflammatory activity to resolve. Therefore it is unlikely that this phenomenon influenced PCa cell homing to the hTEBC^47^.

In conclusion, progress needs to be made to refine the development and characterization of humanized mouse models. In particular, to delineate the specific roles of cells from the TME in PCa growth and metastasis, as well as their persistence in in vivo models, and to investigate the level of humanization required in tissue-engineered bone constructs. The current xenograft approach is a step closer to providing a pertinent platform to study species-specific metastasis of PCa to the bone, which is more clinically relevant than current preclinical models, which do not humanize both primary and secondary TMEs. Once fully developed and characterized, these advanced models will enhance our understanding of human PCa metastasis to bone, as well as allowing the development of a system to test personalized medicine strategies for the treatment of patients with metastatic PCa.

## Methods

### Cell culture

Human osteoblasts (hOBs) were isolated from male patients undergoing hip replacement surgery (Queensland University of Technology Research Ethics approval number 1400001024) by explant culture^48^. The hOBs were cultured in expansion media consisting of minimum essential media alpha (MEM α), 10% fetal bovine serum (FBS) and 100 IU/ml penicillin and 100 µg/ml streptomycin (all Life Technologies, Mulgrave, VIC, Australia), before culture in osteogenic media consisting of expansion media supplemented with 50 µg/ml L-ascorbic acid, 10 mM β-glycerophosphate and 0.1 µM dexamethasone (all Sigma-Aldrich, Castle Hill, NSW, Australia). Patient-derived fibroblasts from radical prostatectomy cancer tissue (CAFs) were isolated and cultured in phenol red-free RPMI-1640 (Life Technologies) supplemented with 10% heat-inactivated FBS, 100 IU/ml penicillin, and 100 µg/ml streptomycin (Life Technologies), 1 nM testosterone (Sigma-Aldrich) and 10 ng/ml basic fibroblast growth factor (bFGF; Life Technologies)^42^. Human prostate-derived CD31 + lymphatic and blood vessel endothelial cells (MVECs) were isolated by enzymatic digestion with 0.25% collagenase II and 0.01% DNase (Worthington, Lakewood, NJ) of fresh radical prostatectomy tissue^14^. The BVECs were seeded into 10 µg/ml fibronectin (Sigma-Aldrich)-coated flasks and both the BVECs and HUVECs were cultured in the Endothelial Cell Growth Medium 2 Kit (EGM2; PromoCell, Heidelberg, Germany). MSCs were cultured in MEM α supplemented with 20% FBS and 100 IU/ml penicillin and 100 µg/ml streptomycin (Life Technologies)^25^. PC-3 cell lines (ATCC, MA, USA) were cultured in 2D and 3D to assess morphology and proliferation. PC-3-Luc1 was stably transduced to express luciferase using a pLenti6/V5-D-TOPO (Life Technologies) lentivirus^4^, and PC-3-Luc2 was obtained from collaborating scientists and routinely cultured in our laboratory. All PC-3 and LNCaP (ATCC) cells were maintained in phenol red-free RPMI-1640 (Life Technologies) supplemented with 10% FBS and 100 U/ml penicillin and 100 µg/ml streptomycin (Life Technologies), referred to below as ‘PCa media’, and used in passages 45, 32 and 14 for PC-3, PC-3-Luc1, and PC-3-Luc2, respectively. LNCaP-luc cells were used at passage 46. PC-3 cells were seeded at 3,000 cells/cm^2^ for 2D experiments and 350,000 cells/cm^3^ for 3D experiments. Cells were cultured for 7 days in 2D and 14 days in 3D. PC-3 and LNCaP cell line authentication was performed by the Genomics Research Center at QUT using short tandem repeat profiling with a ≥80% match for PC-3 (ATCC^®^ CRL-1435™) and LNCaP (ATCC^®^ CRL-1740™), respectively.

### 3D cell encapsulation

Cells were encapsulated in photocrosslinkable semi-synthetic gelatin-methacryloyl (GelMA)-derived hydrogels^49^. A cell suspension of 500,000 cells/ml was prepared in 4% *w/v* GelMA, containing 0.05% *w/v* of a water-soluble photo-initiator (Irgacure, IC2959; BASF, Germany). The precursor cell solution was transferred into a custom-made, rectangular Teflon mold, covered with a glass slide, and crosslinked using a CL-1000 UV cross-linker (UVP Upland, California, USA) with 365 nm wavelength tubes for 12 min (exposed intensity of 2.5 mW/cm^2^ on hydrogel surface). After polymerization, cell-laden hydrogels were removed from the mold, cut into 2 × 4 × 5 mm pieces, and transferred to 24-well plates for culture in PCa media. The media was changed every 3–4 days.

### Microscopy

Microscopy images of GelMA hydrogels were recorded at day 1, 4, and 7 in 2D and day 1, 7, 14 in 3D using a phase-contrast IX73 microscope from Olympus at 10× and 20× magnifications.

### DNA content

For cellular DNA content analysis, the cells were grown in 2D and the cell-laden 3D hydrogels were frozen at −80 °C for at least 48 h after two washes in phosphate buffer saline (PBS). Next, 350 µL of Proteinase K (Invitrogen, Australia) dissolved in phosphate-buffered EDTA (PBE) at 0.5 mg/mL was added to each sample and heated at 60 °C for 12 h using a block heater. The solution was diluted in PBE at a ratio of 1:4 for 2D samples and 1:2 for 3D samples and dispensed in duplicates (100 µL) into black 96-well plates (Corning, Australia). PicoGreen dsDNA quantitation (Invitrogen, Australia) working solution (100 µL) was added. After 5 min of incubation in the dark, the fluorescence (excitation 485 nm, emission 520 nm) was measured using a FLUOstar Omega plate reader (BMG LABTECH, Australia). A standard curve of known λ DNA concentrations ranging from 10 ng/mL to 1 µg/mL was used to calculate the final DNA content of the samples. Data are presented as mean ± SEM, *n* = 3–6.

### Animal experiments

All animal experiments were approved by the Queensland University of Technology Animal Ethics Committee (approval number 130000025) in accordance with the Australian Code of Practice for the Care and Use of Animals for Scientific Purposes. Male NOD-*scid* IL2Rγ^null^ (NOD.Cg - *Prkdc*^*scid*^ *Il2rg*^*tm1Wijl*^ *Hprt*^*b-m3*^/EshJ; NSG) mice were obtained from the Translational Research Institute (TRI) in-house breeding colony at 4–6 weeks of age and acclimatized for up to 3 weeks. Animals were maintained under specific pathogen-free and temperature-controlled conditions. Sterilized food and water were provided *ad libitum* and mice were kept on a 12 h light−dark cycle.

### Intraprostatic injection

A midline skin and peritoneal incision were made on the lower abdomen of male NSG mice. The urinary bladder and seminal vesicles were externalized. A cotton swab was used to further extend the bladder and seminal vesicles to reveal the prostate and perform the intraprostatic injection of the human prostate cells into the dorsal lobe of the mouse prostate. PC-3-Luc or LNCaP-Luc cells (250,000) were injected directly into the mouse prostate with or without accompanying CAFs (200,000) and human BVECs (50,000) in 50 µl of PBS^50^. The bladder and seminal vesicles were replaced and the incisions closed using sutures.

### Bioluminescent imaging (BLI) analysis

Primary tumor formation and cancer cell metastasis were monitored weekly by in vivo BLI using a Xenogen IVIS Spectrum (PerkinElmer, Waltham, MA, USA). Images were acquired 15 min after intraperitoneal injection of 1.5 mg XenoLight D-luciferin potassium salt (PerkinElmer). At the experimental endpoint, the hTEBCs, murine prostate, bones, and organs were excised and analyzed using BLI within 20–30 min of D-Luciferin injection. Signal data were quantified using the Living Image v4.5.2 software (PerkinElmer) using the manual ROI tool to determine the amount of photons emitted for a given time. Signals above 50 counts for the ex vivo analysis were considered positive. The metastatic colonization of different tissues is indicated per mouse, except for the hTEBCs (two constructs per mouse) and kidneys, which are indicated per unit.

### Confocal microscopy imaging

The in vitro tissue-engineered construct was stained with 2 µg/mL fluorescein diacetate (FDA) and 20 µg/mL propidium iodide (PI; both from Life Technologies) to detect living and dead cells, respectively. Additional constructs were fixed overnight at 4 °C in 4% paraformaldehyde (Sigma) then stained with 0.3 U/mL rhodamine 415-conjugated phalloidin and 2.5 µg/mL 4′6-diamidino-2-phenylindole (DAPI; Life Technologies). Samples were imaged with a Leica SP5 confocal scanning laser microscope^49^.

### Scanning electron microscopy (SEM) imaging

For SEM imaging, the in vitro tissue-engineered constructs were fixed with 3% (v/v) glutaraldehyde in 0.1 M sodium cacodylate buffer (pH 7.3) at 4 °C until further processing could be performed. The fixed samples were dehydrated, critical point dried, coated with a gold sputter, and imaged with a Zeiss Sigma VP Field Emission SEM.

### Generation of flat hTEBC

Flat scaffolds of medial grade polycaprolactone (mPCL) were fabricated via melt electrowriting^5^. Scaffolds with a pore size of 250 µm and comprised of 40 micrometer-sized mPCL layers were trimmed to pieces of 4 × 4 mm with a thickness of 400 µm. The scaffolds were coated with calcium phosphate (CaP) by immersion in highly saturated Simulated Body Fluid (SBF 10×) containing 1 M NaCl (ChemSupply, Gillman, SA, Australia), 5 mM KCl, 25 mM CaCl_2_.2H_2_O, 5 mM MgCl_2_.6H_2_O (all from Merck, Darmstadt, Germany) and 10 mM Na_2_HPO_4_ (Sigma-Aldrich), adjusted to pH 6 with NaHCO_3_ (Sigma-Aldrich) for 30 min at 37 °C. The SBF 10× incubation was repeated twice, replacing with fresh SBF 10× each time^51^. The mPCL-CaP scaffolds were seeded with 600,000 hOB/scaffold in 30 µl serum-free MEMα and cultured in well-plates for 2 weeks in expansion media followed by 11 weeks in osteogenic media. Throughout the course of the scaffold culture, hOBs migrated from the scaffold onto the tissue-culture plastic of the well plates and formed a hOB monolayer. The hOB monolayers were cultured together with the scaffold and were allowed to form a hOB cell sheet.

Star-shaped polyethylene glycol (sPEG)-heparin gels were prepared with a total of 100,000 cells (HUVECs:MSCs:hOBs at a ratio of 10:1:1) using gels at a crosslinking degree of γ1.5. The hydrogels were functionalized with a 2:1 molar ratio of RGD to heparin^23^ onto mPCL-CaP scaffolds. The “sandwich” scaffold was assembled with the sPEG-heparin gels cast on an unseeded mPCL-CaP scaffold sheet, layered with an additional sheet of mPCL-CaP scaffold either with or without pre-seeded and cultured hOBs.

### hTEBC implantation procedure

Immediately prior to subcutaneous implantation, the mPCL-CaP in vitro constructs were combined using 60 µl fibrin glue (TISSEEL™ kit, Baxter Healthcare, Australia) embedded with 30 µg rhBMP-7 (Olympus Biotech Corporation, USA). Additionally, the rhBMP-7 and fibrin glue were combined with a hOB cell sheet isolated from the well plates in which the scaffolds were cultured for the hOB laden in vitro constructs. The hOB cell sheet was included as an additional source of hOBs in the hTEBC preparation. The sandwich scaffolds pre-seeded with hOBs were implanted into the left-back of the mouse whereas the unseeded scaffolds were implanted into the right-back of the mouse. To implant the scaffolds, two longitudinal incisions were made on the skin on the left and right back of the mouse. Subcutaneous pockets were created using blunt scissors to gently separate the subcutaneous space. The prepared hTEBCs were inserted into the prepared pockets and the incisions closed with wound closure autoclips (Kent Scientific Corporation, CT, USA). Autoclips were removed by 7−10 days when the surgical site had healed^5^.

### Generation of tubular hTEBC

Tubular mPCL scaffolds with an internal diameter of 5 mm were fabricated via melt electrowriting and CaP-coated as described above^5,7,25^. The mPCL-CaP tubular scaffolds were seeded with 100,000 hOB/scaffold in 30 µl serum-free MEMα and cultured in well-plates for 2 weeks in expansion media followed by 7 weeks in osteogenic media. Throughout the course of the scaffold culture, hOBs migrated from the scaffold onto the tissue-culture plastic of the well plates and formed a hOB monolayer. The hOB monolayers were cultured together with the scaffold and were allowed to form a hOB cell sheet.

A prevascularized niche was generated using 4% GelMA-based hydrogels containing HUVECs and MSCs (10:1 ratio; 5.5 × 10^6^ cells/ml total) 7 days before implantation and pre-cultured in EGM2 (PromoCell, Heidelberg, Germany) supplemented with 125 ng/ml SDF-1α, VEGF and FGF2 (Miltenyi Biotec, NSW, Australia). At the time of implantation, the vascular gel was placed inside the tubular hOB scaffold. The hOB cell sheet was mixed with 30 µl fibrin glue (TISSEEL™ kit, Baxter Healthcare, Australia) and 20 µl of rhBMP-2 (1.5 µg/µl; INFUSE^®^, Medtronic^25^. Two tubular hTEBC were implanted into left and right subcutaneous pockets on the back of male NSG mice as described above.

### Micro CT

Ex vivo analysis was performed on the fixed hTEBCs using a high-resolution µCT scanner (µCT 40, Scanco Medical AG, Switzerland) and scanned at a voxel size of 16 µm. Samples were evaluated at a threshold of 220, a filter width of 0.8, and a filter support of 1. X-ray attenuation was correlated to the sample density with a standard curve generated after scanning hydroxyapatite phantoms with a known mineral density. Bone volume (BV), total volume (TV), and bone volume fraction (BV/TV) were calculated.

### Histology and immunohistochemistry

After necropsy, samples were immediately fixed in 4% paraformaldehyde (Sigma-Aldrich) overnight and then transferred to 80% v/v ethanol until further analysis. Bone samples were decalcified for up to 5 weeks in 10% EDTA (pH 7.4) at 37 °C before all samples were subjected to routine processing and were embedded in paraffin wax. Serial sections were used for hematoxylin and eosin (H&E) staining and immunohistochemistry as outlined in Supplementary Table S6. Endogenous peroxidase activity was quenched with 3% hydrogen peroxide (Sigma-Aldrich) for 15 min and non-specific binding sites were blocked with 2% bovine serum albumin (BSA; Sigma-Aldrich). Primary antibodies were diluted in the blocking buffer. Immunoreactivity was detected using the EnVision + Dual Link System-HRP Rabbit/Mouse kit (Dako, Glostrup, Denmark) and was color developed with liquid diaminobenzidine chromagen (Dako). Sections were counterstained with Mayer’s Hematoxylin (Sigma) before dehydration and mounting. Human tissue and murine tissue were used as positive and negative controls respectively, for validating that antibodies reacted with human and not murine tissues^5^. Images were captured using a Leica SCN400 or a 3D Histech Panoramic high throughput slide scanner.

### Statistics and reproducibility

Data were analyzed using GraphPad Prism v8.1.1 (GraphPad Software, La Jolla, California, USA) and SPSS statistics 23 (IBM Corporation, NSW, Australia). Normally distributed data were analyzed using an unpaired *t*-test, whereas data that were not normally distributed was analyzed using a Mann−Whitney test with a *p*-value < 0.05 accepted as significant. Data are represented as box plots depicting the median, first and third quartile, minimum and maximum, and are overlaid with individual data points. 2D and 3D cell culture data were analyzed using a one-way ANOVA with a Tukey post hoc test with a *p*-value < 0.05 accepted as significant.

### Reporting summary

Further information on research design is available in the Nature Research Reporting Summary linked to this article.

## Supplementary information

Supplementary Information

Description of Additional Supplementary Files

Supplementary Data 1

Reporting Summary

## Data Availability

Data presented in the main figures are available in the Supplementary Data. All other data supporting the findings of this study are available either within the body of the paper, within the Supplementary Information file, or are available from the corresponding authors upon reasonable request.
