# Supplementary material for: A humanized orthotopic tumor microenvironment alters the bone metastatic tropism of prostate cancer cells
Source: Commun Biol. 2021 Aug 30;4:1014. doi: 10.1038/s42003-021-02527-x (PMC8405640; doi:10.1038/s42003-021-02527-x)
Supplement: Supplementary file 1 — Supplementary Information [file 42003_2021_2527_MOESM1_ESM.pdf]

Supplementary Information

A humanized orthotopic tumor microenvironment alters the bone metastatic tropism of prostate cancer cells

Jacqui A McGovern[1,2,3], Nathalie Bock[1,3,4,5], Abbas Shafiee[1,6,7], Laure C Martine[1], Ferdinand Wagner[1,8,9,], Jeremy G Baldwin[1], Marietta Landgraf[1,3], Christoph A. Lahr[1,2], Christoph Meinert[1,7], Elizabeth D Williams[3,4,5], Pamela M Pollock[3,5], Jim Denham[10], Pamela J Russell[3,4,5], Gail P. Risbridger[11], Judith A Clements[3,4,5], Daniela Loessner[1,4,11,12], Boris M Holzapfel[1,4,8], Dietmar W Hutmacher[1,2,3,4,13,*]

[1] Centre in Regenerative Medicine, Queensland University of Technology (QUT), 60 Musk Avenue, Kelvin Grove, QLD 4059, Brisbane, Australia;

[2] School of Mechanical, Medical and Process Engineering (MMPE), Science and Engineering Faculty (SEF), QUT, Brisbane, QLD 4000, Australia;

[3] School of Biomedical Sciences, Faculty of Health, QUT, Brisbane, QLD 4000, Australia

[4] Australian Prostate Cancer Research Centre – Queensland (APCRC-Q), QUT, Princess Alexandra Hospital, Translational Research Institute (TRI), Woolloongabba, QLD 4102, Australia;

[5] Translational Research Institute (TRI), Woolloongabba, QLD 4102, Australia;

[6] UQ Diamantina Institute, Translational Research Institute, The University of Queensland, Brisbane, QLD 4102, Australia;

[7] Herston Biofabrication Institute, Metro North Hospital and Health Service, Brisbane, QLD 4029, Australia;

[8] Musculoskeletal University Centre Munich, Department of Orthopedics and Trauma Surgery, University Hospital Munich, Ludwig-Maximilians University, Campus Großhadern, Marchioninistraße 15, 81377 Munich, Germany;

[9] Department of Pediatric Surgery, Dr. von Hauner Children's Hospital, Ludwig-Maximilians-University of Munich, Lindwurmstraße 4, 80337 Munich, Germany;

[10] School of Medicine and Population Health, University of Newcastle, Callaghan, NSW 2308, Australia;

[11] Department of Anatomy and Developmental Biology, Faculty of Medicine, Nursing and Health Sciences, Monash University, Melbourne, VIC 3800, Australia;

[12] Department of Chemical Engineering and Department of Materials Science and Engineering, Faculty of Engineering, Monash University, Melbourne, VIC 3800, Australia;

[13] ARC Industrial Transformation Training Centre in Additive Biomanufacturing, QUT, 60 Musk Avenue, Kelvin Grove, QLD 4059, Brisbane, Australia.

*Corresponding author

Corresponding author contact information:

Prof. Dietmar W. Hutmacher, PhD, MBA, Chair of Regenerative Medicine, Queensland University of Technology, 60 Musk Avenue, Kelvin Grove, QLD 4059, Australia, Tel: +61 7 31386077, Fax: +61 7 3138 6030, E-mail: dietmar.hutmacher@qut.edu.au
ORCID iD: https://orcid.org/0000-0001-5678-2134

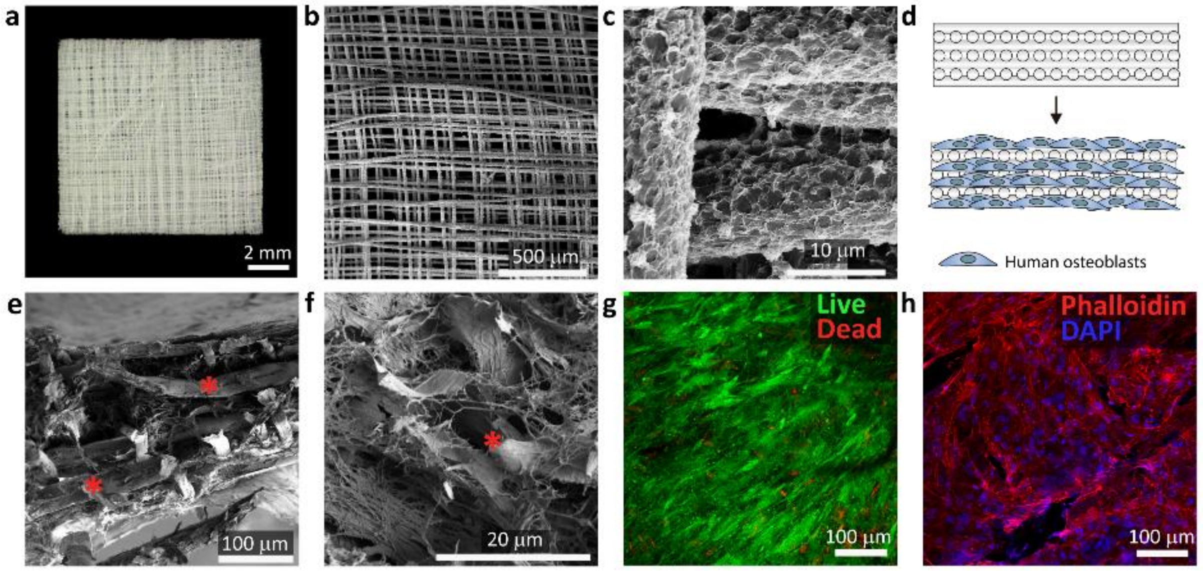

Supplementary Fig. S1. Characterization of the mPCL-CaP scaffold prior to implantation. (a) Representative image of the melt electrospun flat quadratic medical grade polycaprolactone (mPCL) scaffold. Scanning electron microscope (SEM) images of the calcium phosphate (CaP)-coated mPCL scaffold prior to seeding the human osteoblasts (hOBs) were taken at (b) 100X and (c) 5000X magnification to demonstrate the CaP coating. (d) Following CaP-coating, the flat quadratic mPCL-CaP scaffolds were seeded with hOBs and cultured under osteogenic conditions. (e) After 11 weeks of *in vitro* osteogenic culture the hOBs formed a dense cellular and (f) extracellular matrix (ECM) network which infiltrated throughout the scaffold architecture as observed in the cross-sectional SEM images. Scaffold fibers are indicated by the red asterisks. (g) Cellular viability of the hOBs was confirmed using live (fluorescein diacetate, green) -dead (propidium iodide, red) staining and (h) phalloidin (red) and DAPI (blue) to demonstrate that the scaffold contained a dense cell sheet of hOBs.

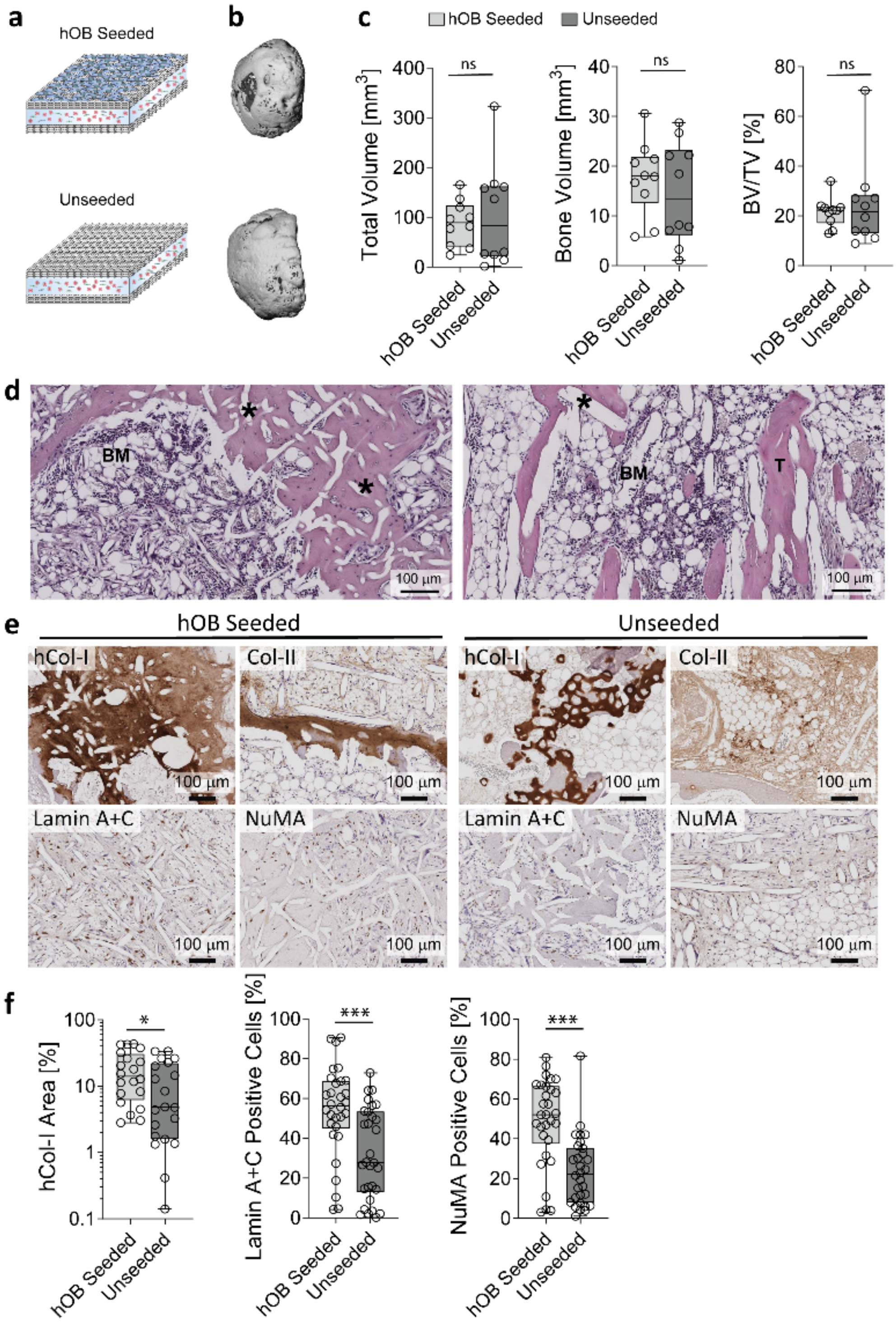

Supplementary Fig. S2. Comparison between human-derived and host-derived microenvironments on ectopic bone formation. (a) Schematic overview of the two different scaffold experimental set-ups; hTEBC populated and pre-cultured with human osteoblasts (hOB seeded) or without osteoblast pre-seeding (unseeded). (b) Micro CT analysis was performed on all hTEBC at the experimental endpoint and (c) the graphs represent total volume (TV), bone volume (BV) and bone volume ratio (BV/TV) of the *ex vivo* constructs. Data are grouped to compare the effect of hOB pre-seeding or unseeded scaffolds on TV, BV and

BV/TV prior to *in vivo* implantation. (d) Histological analysis of the unseeded hTEBC shows that the scaffold formed into a functional organ bone containing marrow (BM), interspersed with calcified trabecula bone tissue (T; right panel). Calcified bone tissue was generated within the unseeded on the scaffolds and formed around the mPCL-CaP fibers (black asterisks). (e) Immunohistochemistry of constructs from both the hOB seeded and unseeded groups were probed for human-derived Type I collagen (hCol-I) and Type II collagen (Col-II), as well as human cells (Lamin A+C and NuMA). Positive immunoreactivity is indicated by the brown staining and sections were counter-stained blue with hematoxylin. (e) Data are grouped to compare the effect of hOB pre-seeding or unseeded scaffolds on human matrix deposition of relative human cell density. hCol-I area (%) was measured in 2 slides at least 50 µm apart from each of the n=10 hTEBC samples (n=20 data points in each group). Lamin A+C and NuMA positive cells were quantified with n=3 regions per slide from each of the n=10 hTEBC per group (n=30 data points per group). Data are represented as box plots depicting the median, first and third quartile, minimum and maximum, and is overlaid with individual data points. TV and BV data were analyzed using an unpaired t test, all other data was analyzed using a Mann-Whitney U Test. *; $P < 0.05$ and ***; $P < 0.001$.

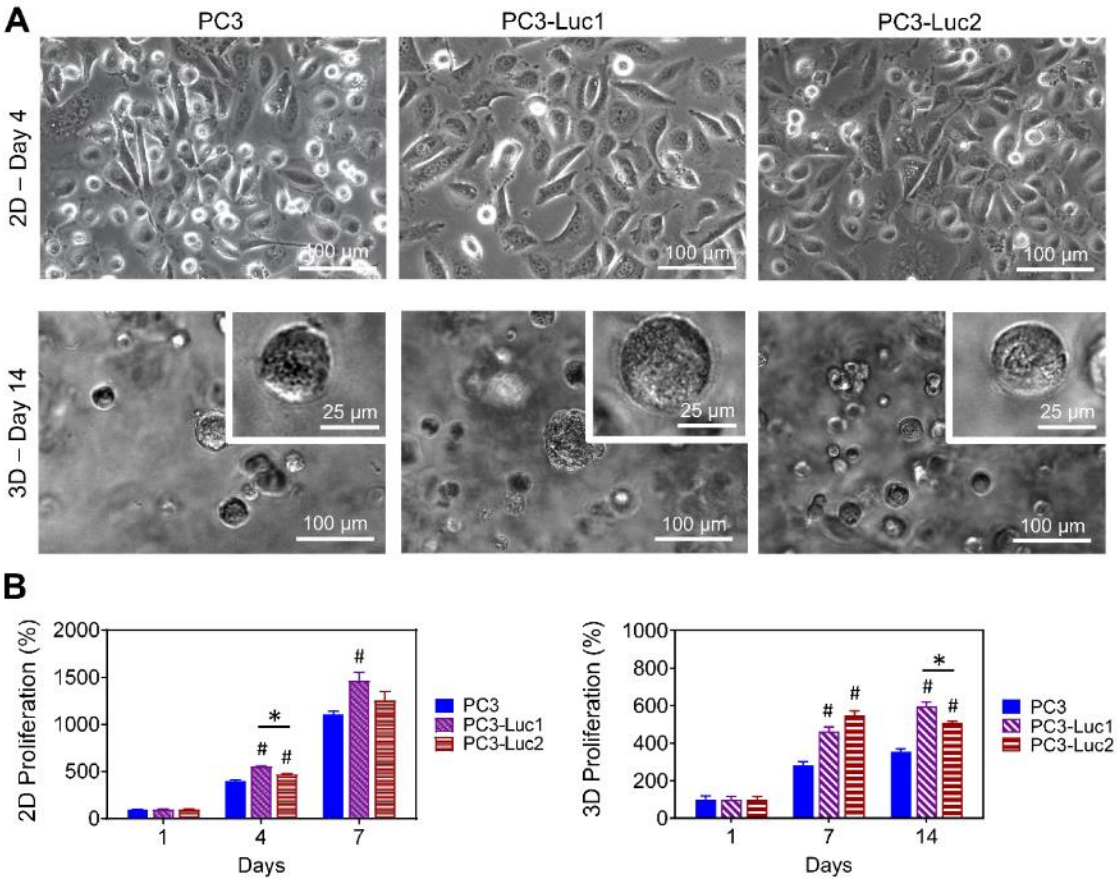

Supplementary Fig. S3. Comparison of the PC-3 cell line growth in 2D and 3D cultures (A) Microscopy images from PC-3 cells (parental or luciferase-expressing) grown on tissue-culture plastic (2D) and in gelatin-methacryloyl (GelMA)-derived hydrogels. B) Cellular DNA content as an indication of PC-3 cell number over time. The data is represented as mean ± SEM, n = 3-6. Statistics: # shows $P < 0.05$ for PC-3-luc1 and PC-3-luc2 compared to PC-3 from the same time point, * shows $P < 0.05$ between PC-3-luc1 and PC-3-luc2. Data was analyzed using a one-way ANOVA with a Tukey post hoc test.

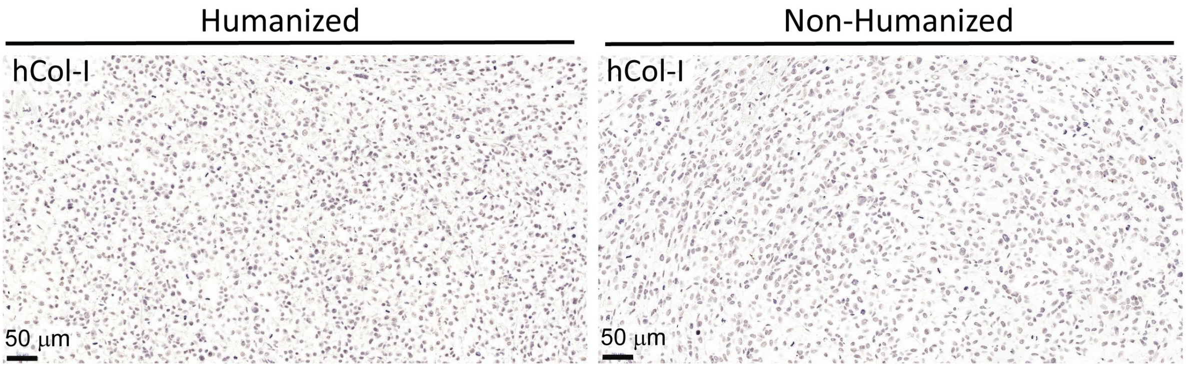

Supplementary Fig. S4. Human Type I collagen is not present in the prostate tumor microenvironment. Prostates were probe for human-derived Type I collagen (hCol-I) using immunohistochemistry. No positive immunoreactivity (as indicated by brown staining) was present in the tissue sections, which were counterstained blue with hematoxylin. The scale bar represents 50 μm.

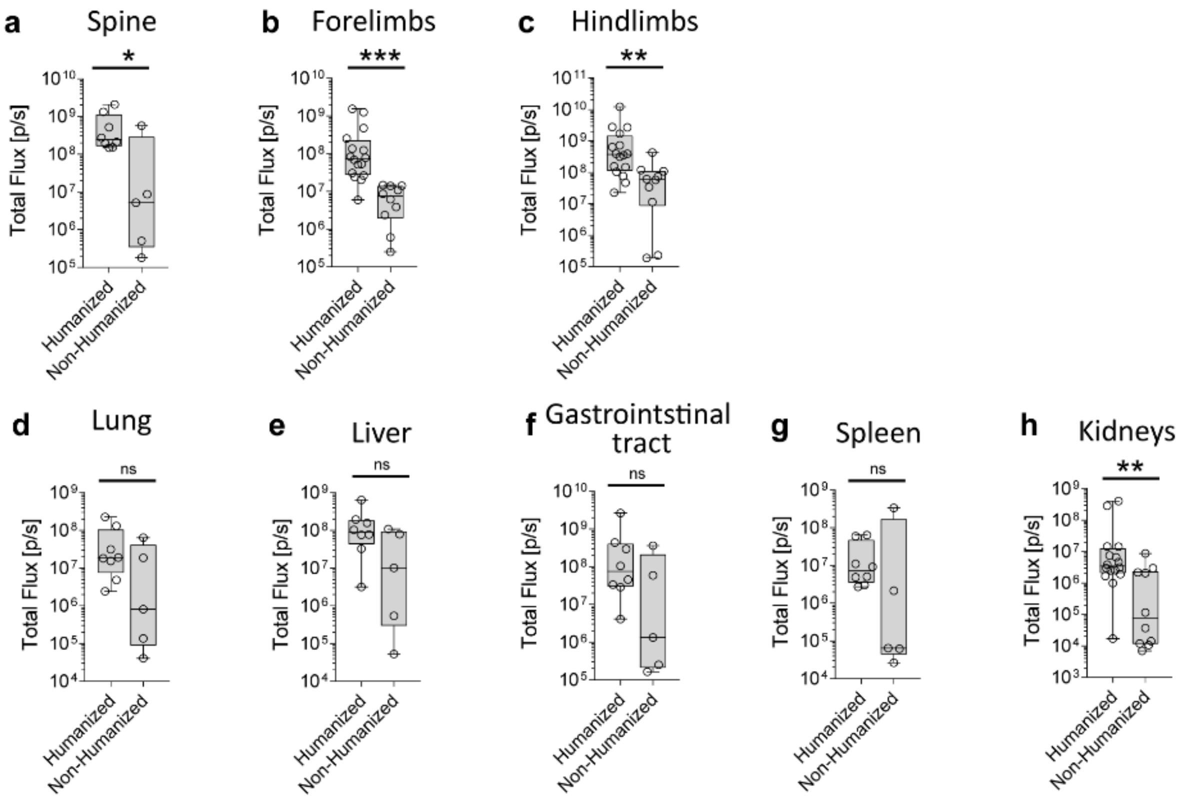

Supplementary Fig. S5. Metastasis of LNCaP-luc cells from the primary prostate tumor to the murine bones and organs. Quantification of the BLI data from LNCaP-luc cells which had metastasized from the primary prostate tumor to the murine (a) spine, (b) forelimbs, and (c) hindlimbs. Quantification of the BLI data from LNCaP-luc cells which had metastasized from the primary prostate tumor to the murine (d) lung, (e) liver, (f) gastrointestinal tract, (g) spleen, and (h) kidneys. Data are represented as box plots depicting the median, first and third quartile, minimum and maximum, and is overlaid with individual data points. All BLI data were not normally distributed and analyzed using a Mann-Whitney U test. N=5 for the non-humanized prostate group and n=8 for the humanized prostate group, except the forelimbs, hindlimbs and kidneys where n=10 and n=16, respectively. *; $P < 0.05$, **; $P<0.01$, and ***; $P < 0.001$.

Supplementary Table S1. PCa cell line take rate in the murine prostate

PCa cell line	Prostate Group	Sample size (n)	PC-3-luc take rate	Average total flux [p/s] (mean±s.d.)
PC-3-luc	Humanized	5	100%	$1.02 \pm 1.11 \times 10^{8}$
	Non-Humanized	5	100%	$6.81 \pm 7.52 \times 10^{7}$
LNCaP-luc	Humanized	8	100%	$3.66 \pm 3.89 \times 10^{10}$
	Non-Humanized	5	100%	$7.63 \pm 10.7 \times 10^{9}$

p/s; photons/second, s.d.; standard deviation

Supplementary Table S2. PC-3-luc metastatic colonization of the hTEBC

Prostate Group	Sample size	Metastatic Colonization[#] per hTEBC (hOB seeded)	Metastatic Colonization[#] hTEBC (Unseeded)	Metastatic Colonization[#] (all hTEBC)	Average total flux [p/s] (mean±s.d.)
Humanized	10 (5 mice)	60%	80%	70%	$5.6 \pm 14.9 \times 10^{5}$
Non-Humanized	10 (5 mice)	80%	60%	70%	$1.17 \pm 0.94 \times 10^{5}$

p/s; photons/second, s.d.; standard deviation, [#] Metastatic colonization defined as positive on bioluminescence imaging

Supplementary Table S3. PC-3-luc metastatic colonization of the mouse bone and organs

	Prostate Group	Sample size (n)	Metastatic colonization# per mouse	Average total flux [p/s] (mean±s.d.)
Spine	Humanized	5	100%	$3.43 \pm 4.66 \times 10^6$
	Non-Humanized	5	100%	$1.64 \pm 1.75 \times 10^6$
Forelimbs	Humanized	10	20%	$3.10 \pm 3.79 \times 10^4$
	Non-Humanized	10	70%	$2.39 \pm 1.52 \times 10^4$
Hindlimbs	Humanized	10	90%	$1.48 \pm 2.0 \times 10^5$
	Non-Humanized	10	70%	$6.60 \pm 5.39 \times 10^4$
Lungs	Humanized	5	100%	$3.96 \pm 3.1 \times 10^6$
	Non-Humanized	5	100%	$1.80 \pm 1.20 \times 10^6$
Liver	Humanized	5	100%	$6.57 \pm 12.0 \times 10^6$
	Non-Humanized	5	100%	$5.09 \pm 8.36 \times 10^6$
GI tract	Humanized	5	100%	$5.81 \pm 7.45 \times 10^7$
	Non-Humanized	5	100%	$1.33 \pm 1.65 \times 10^7$
Spleen	Humanized	5	100%	$5.47 \pm 5.36 \times 10^6$
	Non-Humanized	5	80%	$1.54 \pm 1.80 \times 10^6$
Kidneys	Humanized	10	80%	$2.13 \pm 3.58 \times 10^6$
	Non-Humanized	10	90%	$4.60 \pm 5.93 \times 10^5$

p/s; photons/second, s.d.; standard deviation, # Metastatic colonization defined as positive on bioluminescence imaging

Supplementary Table S4. LNCaP-luc metastatic colonization of the hTEBC

Prostate Group	Sample size	Metastatic Colonization[#] per mouse	Average total flux [p/s] (mean±s.d.)
Humanized	16 (8 mice)	87.5%	$2.18 \pm 4.47 \times 10^{6}$
Non-Humanized	10 (5 mice)	40%	$1.07 \pm 1.77 \times 10^{5}$

p/s; photons/second, s.d.; standard deviation, [#] Metastatic colonization defined as positive on bioluminescence imaging

Supplementary Table S5. LNCaP-luc metastatic colonization of the mouse bone and organs

	Prostate Group	Sample size (n)	Metastatic colonization# per mouse	Average total flux [p/s] (mean±s.d.)
Spine	Humanized	8	100%	$6.13 \pm 7.12 \times 10^{8}$
	Non-Humanized	5	100%	$1.17 \pm 2.53 \times 10^{8}$
Forelimbs	Humanized	16	100%	$2.65 \pm 4.62 \times 10^{8}$
	Non-Humanized	10	100%	$7.58 \pm 5.70 \times 10^{6}$
Hindlimbs	Humanized	16	100%	$1.43 \pm 3.03 \times 10^{9}$
	Non-Humanized	10	100%	$8.97 \pm 12.6 \times 10^{8}$
Lungs	Humanized	8	100%	$5.63 \pm 8.05 \times 10^{7}$
	Non-Humanized	5	100%	$1.68 \pm 2.77 \times 10^{7}$
Liver	Humanized	8	100%	$1.61 \pm 2.04 \times 10^{8}$
	Non-Humanized	5	100%	$3.98 \pm 5.09 \times 10^{7}$
GI tract	Humanized	8	100%	$4.52 \pm 9.05 \times 10^{8}$
	Non-Humanized	5	100%	$8.50 \pm 15.8 \times 10^{7}$
Spleen	Humanized	8	100%	$2.00 \pm 1.61 \times 10^{7}$
	Non-Humanized	5	100%	$6.81 \pm 15.1 \times 10^{7}$
Kidneys	Humanized	10	93.75%	$4.74 \pm 11.8 \times 10^{7}$
	Non-Humanized	10	70%	$1.62 \pm 2.77 \times 10^{6}$

p/s; photons/second, s.d.; standard deviation, # Metastatic colonization defined as positive on bioluminescence imaging

134 **Supplementary Table S6. Antibodies and antigen retrieval for Immunohistochemistry**

Antibody	Company	Product code	Antigen Retrieval	Incubation
Lamin A+C#	Abcam	ab108595	Tris-EDTA Buffer+0.1% Tween-20, pH 9.0 (95 °C/5 min)	1:300, 1 hr at RT
Nuclear mitotic apparatus protein 1 (NuMA)#	Abcam	ab97585	Tri-sodium Citrate Buffer+0.1% Tween-20, pH 6.0 (95 °C/5 min)	1:200, 1 hr at RT
CD44#	DSHB	H4C4	Tri-sodium Citrate Buffer+0.1% Tween-20, pH 6.0 (95 °C/5 min)	1:50, overnight at 4 °C
Type I collagen (hCol-I)#	Abcam	ab138492	Tris-EDTA Buffer+0.1% Tween-20, pH 9.0 (95 °C/5 min)	1:500, 1 hr at RT
Type II collagen (Col-II)	DSHB	II-II6B3	Proteinase K, 15 min at RT	1:200, overnight at 4 °C
Osteocalcin (hOCN)#	Abcam	ab13420	Proteinase K, 15 min at RT	1:200, overnight at 4 °C
Prostate specific membrane antigen (PSMA)	Abcam	ab133579	Tri-sodium Citrate Buffer+0.1% Tween-20, pH 6.0 (95 °C/5 min)	1:500, 1 hr at RT

135 #These antibodies react with human cells/tissues, but not with mouse cells/tissues when probed using immunohistochemistry.
