# Supplementary material for: A humanized orthotopic tumor microenvironment alters the bone metastatic tropism of prostate cancer cells
Source: Commun Biol. 2021 Aug 30;4:1014. doi: 10.1038/s42003-021-02527-x (PMC8405640; doi:10.1038/s42003-021-02527-x)
Supplement: Supplementary file 2 — Description of Additional Supplementary Files [file 42003_2021_2527_MOESM2_ESM.pdf]

Description of Additional Supplementary Files

File Name: Supplementary Data 1

Description: Source data for graphs:

Fig 2D – hOB seeded hTEBC (micro-CT analysis)
Fig 2G – hOB seeded hTEBC NuMA positive cells
Fig 2H – hOB seeded hTEBC LaminA+C positive cells
Fig 3C – In vivo BLI time course (PC-3 mice)
Fig 3D – Ex vivo BLI PC-3 prostates
Fig 3F – PC-3 prostate NuMA positive cells
Fig 3G – PC-3 prostate LaminA+C positive cells
Fig 4C – In vivo BLI time course (LNCaP mice)
Fig 4E – Ex vivo BLI LNCaP prostates
Fig 4H – Ex vivo BLI LNCaP hTEBC
Fig 5C – Ex vivo BLI PC-3 hTEBC (all)
Fig 5D – Ex vivo BLI PC-3 hTEBC (hOB seeded)
Fig 5E – Ex vivo BLI PC-3 hTEBC (Unseeded)
Fig 5G – Ex vivo BLI PC-3 (murine bones)
Fig 5H – Ex vivo BLI PC-3 (murine visceral organs)
